# Multipass Friction Stir Processing of Laser-Powder Bed Fusion AlSi10Mg: Microstructure and Mechanical Properties

**DOI:** 10.3390/ma16041559

**Published:** 2023-02-13

**Authors:** Akbar Heidarzadeh, Mahsa Khorshidi, Roghayeh Mohammadzadeh, Rasoul Khajeh, Mohammadreza Mofarrehi, Mousa Javidani, X.-Grant Chen

**Affiliations:** 1 Department of Materials Engineering, Azarbaijan Shahid Madani University, Tabriz P.O. Box 53714-161, Iran; 2 School of Metallurgy and Materials Engineering, Iran University of Science and Technology, Tehran P.O. Box 16846-13114, Iran; 3 Department of Applied Science, University of Quebec at Chicoutimi, Saguenay, QC G7H 2B1, Canada

**Keywords:** AlSi10Mg alloy, laser-powder bed fusion, friction stir processing, microstructure, mechanical properties

## Abstract

The effect of multipass friction stir processing (FSP) on the microstructure and mechanical properties of an AlSi10Mg alloy produced by laser-powder bed fusion was investigated. FSP was performed at a rotational speed of 950 rpm and traverse speed of 85 mm/min. The results indicated that FSP destroyed the coarse grain structure in the as-built AlSi10Mg by generating fine and equiaxed grain structures with shear texture components of $A_{1}^{*}\left(111\right)\left[\bar{1}\bar{1}2\right]$ and $A_{2}^{*}\left(111\right)\left[11\bar{2}\right]$, in addition to causing fragmentation and refinement of the Si networks. FSP reduced the tensile strength slightly but significantly improved ductility. One-pass FSP exhibited superior mechanical properties compared with the two- and three-pass scenarios. The higher strength of the one-pass sample was attributed to the strengthening mechanisms induced by the Si particles, which were grown by repeated FSP. The higher ductility of the one-pass sample was explained using the kernel and grain average misorientations. Furthermore, the post-FSP microstructural evolution and fracture behavior of the samples were discussed.

## 1. Introduction

Laser-powder bed fusion (L-PBF), also termed selective laser melting, is an additive manufacturing method and a rapid three-dimensional (3D) production technique that can be applied to a wide range of metals and alloys [1]. Recently, several studies have focused on L-PBF-related topics, looking at how they overcome the restrictions of traditional manufacturing methods, such as machining and casting [2]. From a metallurgical point of view, L-PBF possesses characteristics similar to those of laser welding; however, it includes higher cooling rates. During L-PBF, in accordance with the 3D geometry, a laser (single or multiple beams) selectively scans and melts a layer of powder that was previously laid on a stage. Another layer of powder is subsequently deposited on the stage, and the process is repeated for the new layer. By repeating this procedure, a metal part is produced until the entire 3D geometry is scanned [3].

The AlSi10Mg alloy, a common L-PBF alloy owing to its high printability, has recently attracted research attention in the field of industrial engineering [4]. For example, the characteristics of L-PBF AlSi10Mg, such as its high strength-to-density ratio, make it an appropriate candidate for aerospace applications. However, AlSi10Mg produced by L-PBF has the disadvantage of low ductility owing to the presence of porosity and inhomogeneity in its microstructure [4,5]. Therefore, several studies have explored new methods for improving the ductility of L-PBF AlSi10Mg [4,5].

Among various post-heat and post-deformation treatments, friction stir processing (FSP) has been introduced as a promising method of increasing the ductility of L-PBF AlSi10Mg [4]. FSP, which is based on the friction stir welding (FSW) concept, is a solid-state deformation method that can be applied to modify the microstructure and mechanical properties of metals and alloys [6]. During FSP, the deformation and heat induced by a non-consumable-rotating tool cause various microstructural evolutions, such as dynamic recrystallization (DRX), which destroys the initial microstructure of the material [5,7]. The final microstructure and mechanical properties of friction stir processed (FSPed) metals and alloys, such as L-PBF AlSi10Mg, depend on process parameters such as tool rotational speed, tool traverse speed, tool geometry, and tool axial force [8]. 

Yang et al. [9] investigated the effect of tool rotational speed (750 and 1180 rpm, at a constant tool traverse speed of 37.5 mm/min) on the microstructure and mechanical properties of L-PBF AlSi10Mg. They determined that an increase in tool rotational speed results in higher density by eliminating gas and solidification porosities inside the base material (BM). In addition, they confirmed that FSP at an optimal tool rotational speed can improve tensile elongation by 298%. Maamoun et al. [10] employed FSP for the surface processing of an L-PBF AlSi10Mg disk using a tool rotational speed of 1750 rpm and a tool traverse speed of 160 mm/min. They concluded that FSP is a promising method for localized modification of the microstructure and mechanical properties of L-PBF AlSi10Mg alloys. Zhao et al. [11,12] and Macías et al. [13] studied the effect of FSP on the damage mechanisms in L-PBF AlSi10Mg, using a rotational speed of 1000 rpm and a traverse speed of 500 mm/min. They determined that the FSP increases ductility by globularization of Si in the microstructure, which delays the formation of voids on Si particles and hinders void growth in the matrix. Rafieazad et al. [14] employed FSP to enhance the corrosion resistance of L-PBF AlSi10Mg. They demonstrated that FSP modifies the corrosion properties by forming a uniform microstructure, including a uniform distribution of Si particles, fine and equiaxed grains, and a small fraction of low-angle grain boundaries (LAGBs). 

The number of passes plays a key role in determining the final properties of FSPed metals and alloys because of their direct effect on microstructural evolution, which has been investigated by several studies on different types of material, including aluminum-based cast alloys [15]. For example, Nakata et al. [16] studied the effect of multipass FSP on the microstructural and mechanical properties of a die-cast aluminum alloy. They reported that multipass FSP causes grain refinement, elimination of casting defects, and uniform distribution of Si particles in the matrix, thereby improving the tensile strength compared to that of the BM. Moharrami et al. [17] employed multipass FSP on Mg_2_Si-rich aluminum alloys. They observed that, by increasing the number of passes, more Mg_2_Si fragmentation and grain refinement can be achieved. Hence, the hardness and strength of the processed alloy improved, whereas the wear rate and friction coefficient deteriorated. Sing et al. [18] investigated the influence of multipass FSP on the wear properties and machinability of an Al–Si hypoeutectic A356 alloy. They reported that highest wear resistance and machinability can be achieved by employing two-pass FSP. 

The results from these studies indicate that multipass FSP has never been implemented on L-PBF AlSi10Mg, which can affect the microstructure and corresponding mechanical properties. Therefore, the aim of this study was to explore the effect of multipass FSP (a total of three passes) on the characteristics (mainly microstructure evolution and mechanical properties) of L-PBF AlSi10Mg alloys.

## 2. Materials and Methods

**Figure 1:**
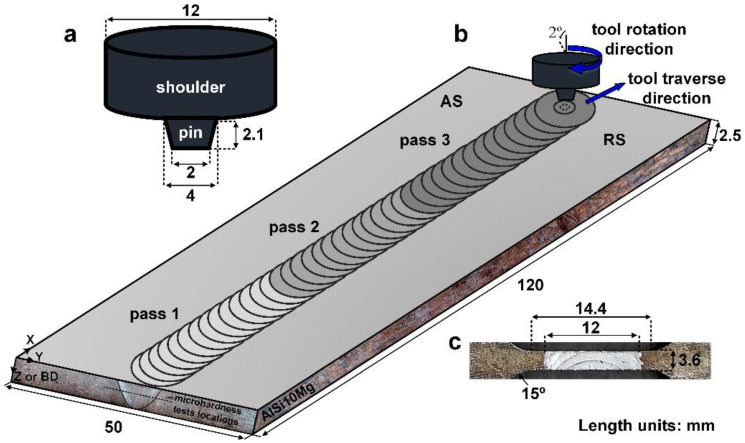
Schematics of (**a**) FSP tool, (**b**) FSP process, and (**c**) tensile test samples.

The AlSi10Mg (10 wt.% Si, 0.4 wt.% Mg, and 89.6 wt.% Al) plate with dimensions of 120 × 50 × 2.5 mm was produced by L-PBF using a Noura M100P machine (Noura, Isfahan, Iran) within the following parameters: number of slices, 260; powder consumption, 80 g; build time, 2 h; layer thickness, 30 µm; energy density, 60 J/mm^3^; hatch space, 0.19 mm; and scan rotation, 67°. Gas-atomized AlSi10Mg powder with an average diameter of <65 µm was used. After L-PBF, multipass FSP was used for post-processing of the AlSi10Mg plate using three passes, denoted as samples S1, S2, and S3. To achieve this, FSP was performed perpendicular to the building direction (BD) of the plate at a rotational speed (*W*) of 950 rpm, traverse speed (*V*) of 85 mm/min, plunge depth of 0.1 mm, and tilt angle of 2° using an H13 steel tool. The FSP tool was composed of a pin and shoulder with dimensions, as schematically shown in Figure 1a. A schematic of the multipass FSP used in this study is shown in Figure 1b. In order to save processing time, the length of the plate was divided into three parts. The first pass was conducted along the entire length of the plate, which took 1.77 min. The second pass was performed on two-thirds of the plate’s length for 0.8 min. The third pass was conducted on the final third of the plate for 0.33 min. Considering 10 s (0.16 min) of shoulder-holing time at the start of each pass, the processing time of three passes totaled 3.38 min. 

The macrostructure and microstructure of the joint were characterized using optical microscopy, scanning electron microscopy (SEM) equipped with an electron backscatter diffraction (EBSD) unit, and X-ray diffraction (XRD). Metallographic samples were cut from the joint perpendicular to the FSW direction, polished, and etched with a solution of HNO_3_ (5 mL), HCl (3 mL), HF (2 mL), and distilled water (190 mL). EBSD analysis was performed in the SEM with a step size of 0.2 µm, and all corresponding data (that is, inverse pole figure (IPF), grain boundary (GB), grain average misorientation (GAM), and Taylor factor maps) were processed using TSL-OIM software. To identify the grain and sub-grain structures, the boundaries and sub-boundaries were defined based on the misorientation angle as low-angle boundaries (LAGBs: <15°) and high-angle boundaries (HAGBs: >15°). Tensile tests were performed to determine the mechanical properties of the joints and BM. The tensile samples were wire cut according to the JIS no. 7 standard (Figure 1c) and tested at a strain rate of 1 mm/min. Moreover, the fractured surfaces of the tensile samples were characterized using SEM.

## 3. Results

### 3.1. Microstructural Zones

**Figure 2:**
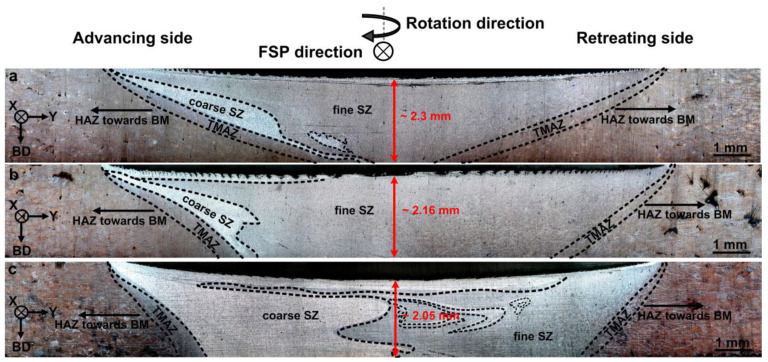
Cross-sectional macrostructure of (**a**) S1, (**b**) S2, and (**c**) S3.

**Figure 3:**
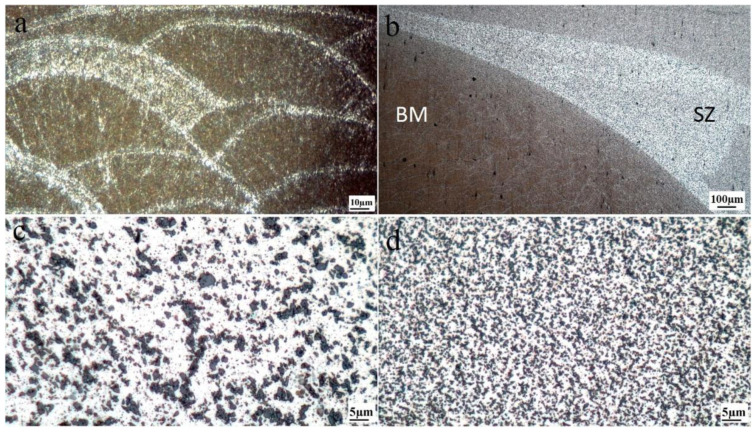
Optical microscopy images showing typical microstructural zones S1: (**a**) BM, (**b**) transition zone between SZ and BM including TMAZ and HAZ, (**c**) coarse SZ, and (**d**) fine SZ. The fine and coarse SZ refer to the zones with fine and coarse Si particles.

The cross-sectional macrostructures of the different FSPed samples are shown in Figure 2, indicating the presence of distinct zones including the BM, transition zone, and stir zone (SZ). It is obvious from the macrostructure images (Figure 3a) that the BM is not affected by heat and deformation during FSP, exhibiting a typical microstructure of L-PBF AlSi10Mg containing overlapping melt pool boundaries (MPBs). However, the transition zone between the BM and SZ (Figure 3b), which is composed of the heat-affected (HAZ) and thermomechanically affected zones (TMAZ), is influenced during FSP. By reaching the center of the SZ, the temperature, strain, and strain rate induced by the rotational tool increased to their maximum values. Hence, the microstructural evolution can be completed in the SZ, whereas it only partially occurs in the transition zone owing to inadequate heat and deformation.

From Figure 2, based on the size of the Si particles, the SZ can be divided into two regions: coarse and fine SZs. The coarse SZ is found to be created in the advancing side (between TMAZ and fine SZ) and under the shoulder, where the temperature is higher than other regions [7]. Moreover, the size of the coarse SZ area is increased by multipass FSP (Figure 2). This can be related to the repeated heat input derived from multipass FSP. The microstructures of the coarse and fine SZs (for example in S1) are shown in Figure 3c,d, indicating higher growth of Si particles in the coarse SZ than the fine SZ. As shown in Figure 2, the width and depth of the FSP zone was increased by the multipass FSP. Furthermore, the large L-PBF porosities were eliminated after FSP (Figure 2). The peak temperature in SZ during FSP directly affects the microstructure of the corresponding zones, which depends on the rotational and traverse speeds based on the following equation [6,7]:

(1) $$T=KT_{m}{\left(\frac{W^{2}}{10^{4}\times V}\right)}^{\alpha }$$

where $T_{m}$ stands for the melting point of the AlSi10Mg (~600 °C), and *K* and *α* are the constants in the ranges of 0.04–0.06 and 0.65–0.75, respectively. By inserting the corresponding values to Equation (1), the temperature in SZ was estimated to be ~315 °C, an appropriate temperature for the occurrence of restoration and dynamic softening mechanisms in aluminum alloys [6]. Therefore, the material can flow easily during FSP to fill the porosities. 

**Figure 4:**
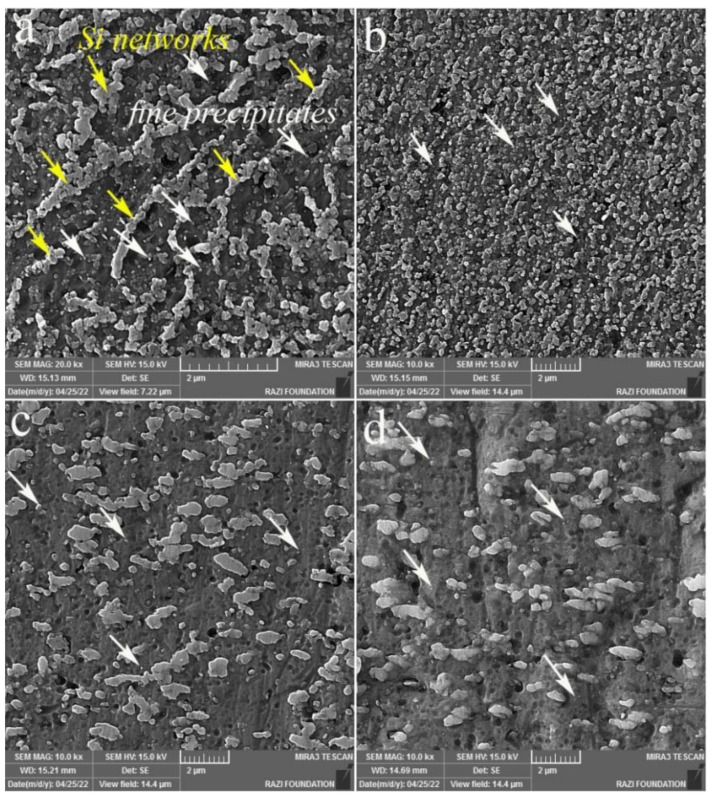
Secondary electron SEM images: (**a**) Si networks in BM, and (**b**–**d**) Si particles at the center of the SZs (fine SZs) for S1, S2, and S3, respectively. The yellow arrows in (**a**) indicate the fibrous Si networks. The white arrows denote the fine precipitates.

The SEM micrographs of the melt pool interior (Figure 3) and fine SZs are shown in Figure 4, which confirm the fragmentation of fibrous Si networks (indicated by yellow arrows in Figure 4a) into individual particles (Figure 4b–d) by FSP. In addition, one-pass FSP (S1) caused the finest Si particles among the three FSPed samples, whereas there was no considerable difference between S2 and S3. Importantly, from Figure 4, the presence of fine Si precipitate in the BM is observed (white arrows in Figure 4a in the Al matrix surrounded by Si networks), which can also be found in the SZs (Figure 4b–d).

**Figure 5:**
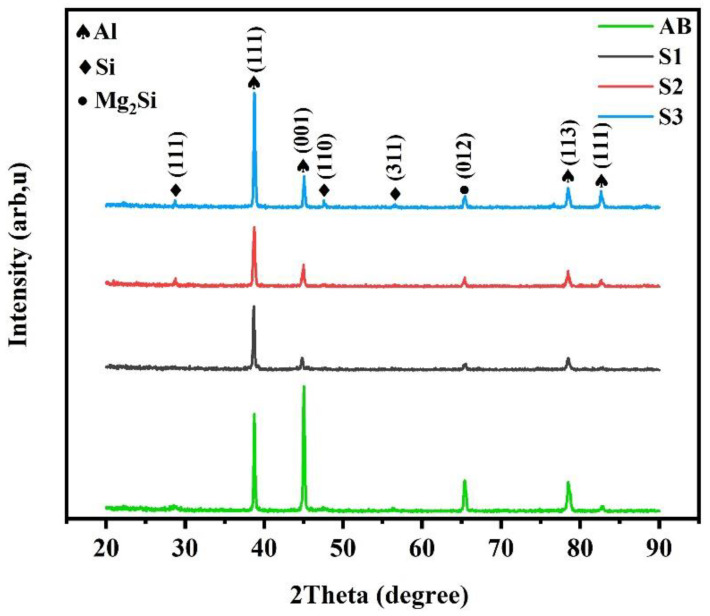
XRD patterns for BM (as-built material: AB) and SZ of different FSPed samples.

The XRD patterns (Figure 5) indicate the existence of Mg_2_Si precipitates in the BM and SZs in addition to the Si phase, which is well-described in literature [4]. Moreover, the change in (111) Al peak intensity suggests the texture formation during FSP, which is discussed in detail based on EBSD pole figures and inverse pole figures.

### 3.2. EBSD Maps

**Figure 6:**
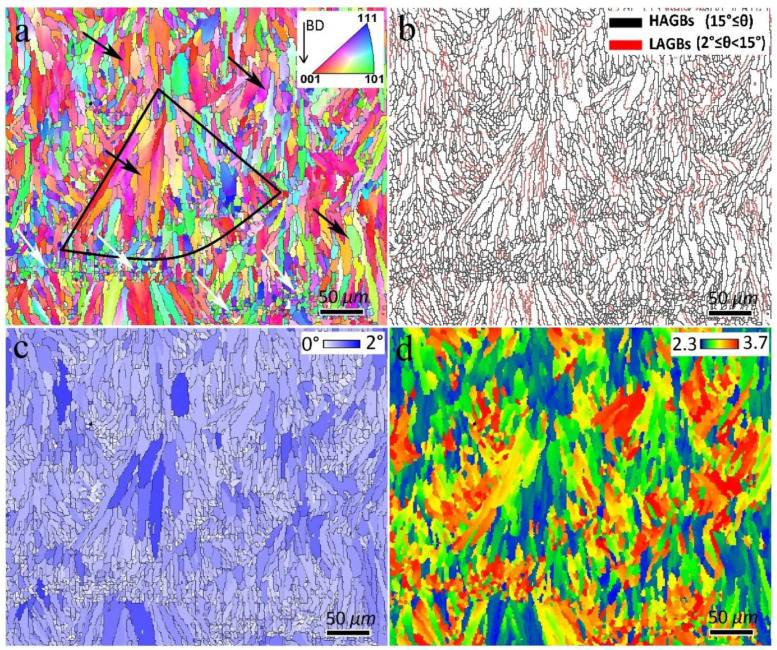
(**a**) Inverse pole figure (IPF), (**b**) Grain boundary (GB), (**c**) Grain average misorientation (GAM), and (**d**) Taylor factor maps of BM. In (**a**,**c**), HAGBs are superimposed as black boundaries. In (**d**), the {111}<110> family of slip systems was considered for calculation of Taylor factor values.

As shown in Figure 6a, the BM is composed of two types of grain, including large elongated grains (black arrows) and fine equiaxed grains (white arrows) within a single melt pool, as indicated by the black line area. The average grain size of the BM was 15.8 µm. The elongated grains are mainly formed parallel to the BD, going against the heat transfer direction during the solidification of the melt pools [4]. The formation of fine equiaxed grains occurs when the melt pool is consumed by elongated grains and the temperature gradient decreases to a certain value for the nucleation of equiaxed grains [19]. From Figure 6b, the BM contained 81% and 19% HAGBs and LAGBs, respectively. The grain average misorientation (GAM) map (Figure 6c) for misorientation between 0° and 2° can be employed to qualitatively estimate the dislocation density, with higher GAM values indicating a higher dislocation density in the material [20]. As shown in Figure 6c, in the BM, the elongated grains have larger GAM values than the fine equiaxed grains, and the average GAM value was calculated as 0.53°. The Taylor factor in polycrystalline metals and alloys represents the effect of crystallographic texture on yield strength [21]. A higher Taylor factor indicates higher strength of the metals and alloys. In Figure 6d, the Taylor factor map of the BM is shown, in which the average value of the Taylor factor was calculated to be 2.84.

**Figure 7:**
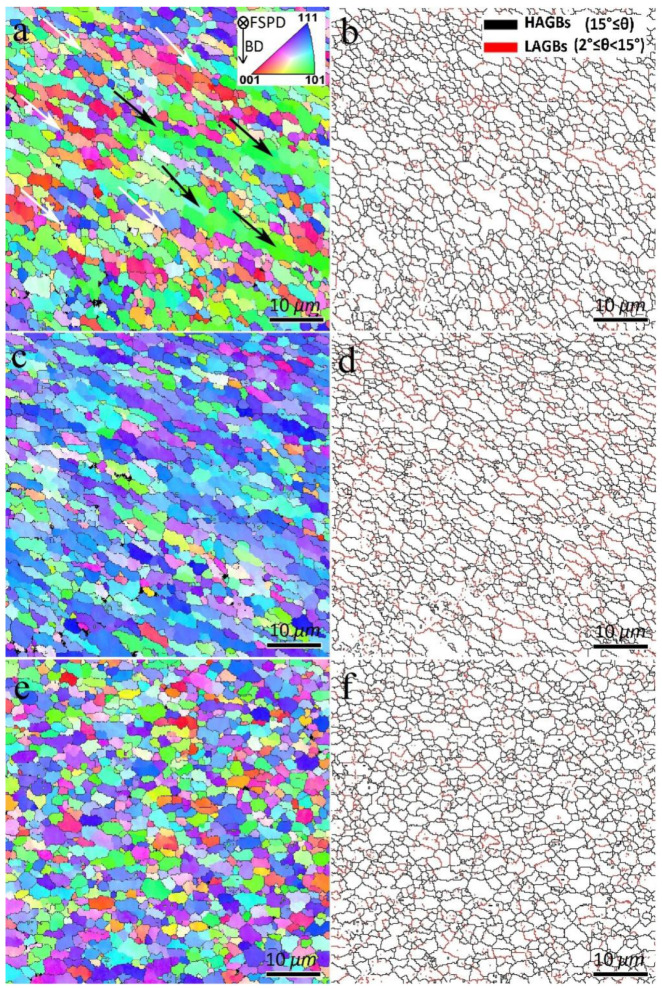
Inverse pole figure (IPF) and grain boundary (GB) maps of SZs: (**a**,**b**) S1, (**c**,**d**) S2, and (**e**,**f**) S3.

**Figure 8:**
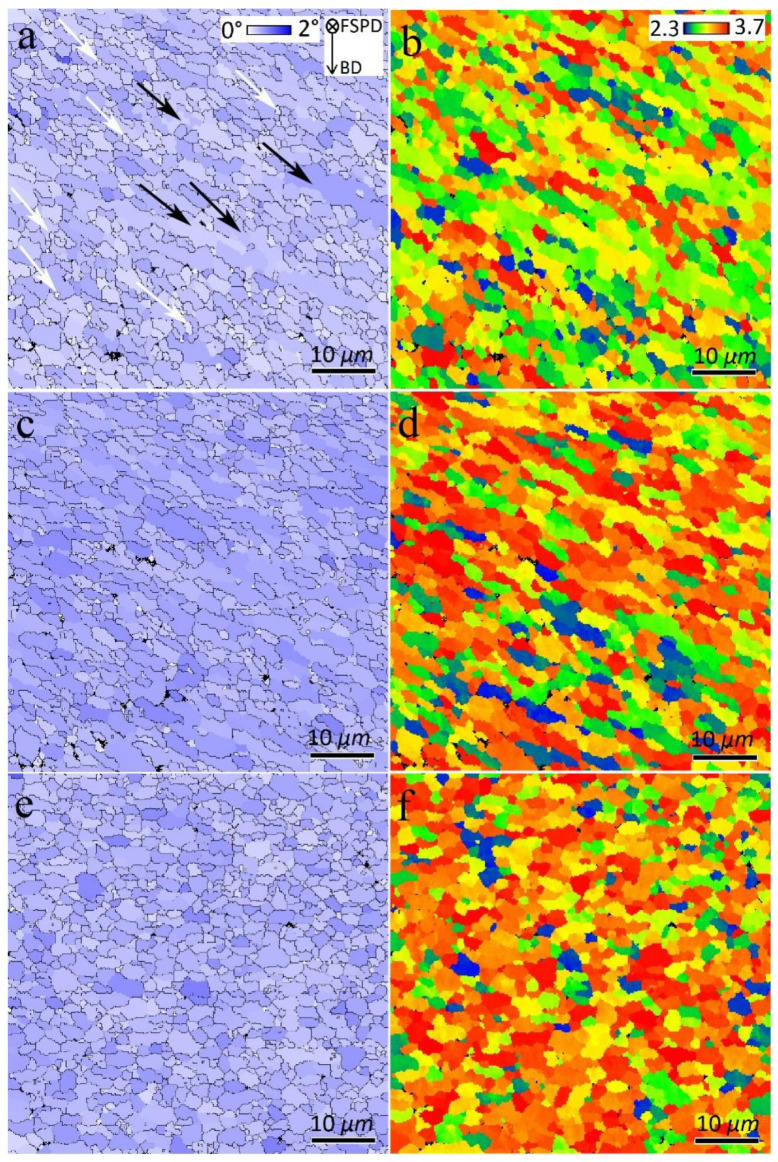
Grain average misorientation (GAM) and Taylor factor maps of SZs: (**a**,**b**) S1, (**c**,**d**) S2, and (**e**,**f**) S3.

The EBSD maps of S1, S2, and S3 SZs are shown in Figure 7 and Figure 8. From Figure 7, FSP leads to grain refinement in the SZs, and the average grain sizes of S1, S2, and S3 were calculated as 3.0, 3.1, and 2.5 µm, respectively. The formation of fine-grained structures in the SZs of FSPed metals and alloys is related to the occurrence of different DRX mechanisms [6]. According to the GB map of the samples in Figure 7, a large number of grain boundaries (80%, 71%, and 83% for S1, S2, and S3, respectively) have the characteristics of HAGBs. 

The GAM maps of the FSPed samples in Figure 8 and their calculations indicated 0.45°, 0.59°, and 0.54° values for S1, S2, and S3, respectively. Therefore, the first FSP pass resulted in reduced dislocation density. However, more passes of FSP resulted in larger GAM values than those of the BM (Figure 6). Furthermore, from Figure 7 and Figure 8, the formation of a bimodal grain structure in S1 is evident, including fine grains (white arrows in Figure 7 and Figure 8) and elongated large grains (black arrows in Figure 7 and Figure 8), which decreased in S2 and disappeared in S3. According to the GB maps in Figure 7 and the GAM maps in Figure 8, the larger grains in the bimodal structures of S1 and S2 contained substructures such as LAGBs and higher dislocation densities compared to those of sample S3. Analysis of the Taylor factor maps and values (Figure 8) indicated that the bimodal structure was enhanced compared to that of the BM. The Taylor factor values for S1, S2, and S3 are calculated as 2.98, 3.12, and 3.18, respectively.

### 3.3. Texture

**Figure 9:**
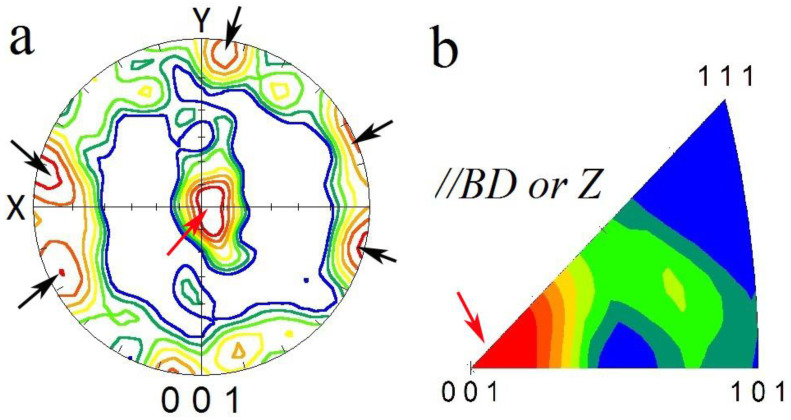
(001) pole figure (**a**) and (001) inverse pole figure (**b**) of BM. X and Y in (**a**) belong to the BM coordination system as indicated in Figure 1b. BD denotes the building direction of the BM during L-PBF. Inverse pole figure in (**b**) was generated parallel to BD.

The (001) pole figure (PF) and inverse pole figure (IPF) along the [001] direction of L-PBF AlSi10Mg are shown in Figure 9. It presents the strong texture formation in which the [001] crystallographic direction is aligned with the BD, indicated by red arrows at the center and black arrows at the periphery of PF. The formation of such a texture component is related to the solidification nature of metals and alloys with face-centered cubic (FCC) crystallographic structures. During the solidification of these alloys, the preferred growth directions (<100> directions) are aligned parallel and opposite to the heat transfer direction (that is, //BD) in the melt pools, hence a <100>//BD can be formed (Figure 9b). Texture weakening may occur due to the thermal gradient that is not parallel to the BD, and, depending upon the L-PBF parameters, may have a radial nature [19]; hence, the elongated grains (Figure 6) can be tilted outwards toward the center of the melt pools.

**Figure 10:**
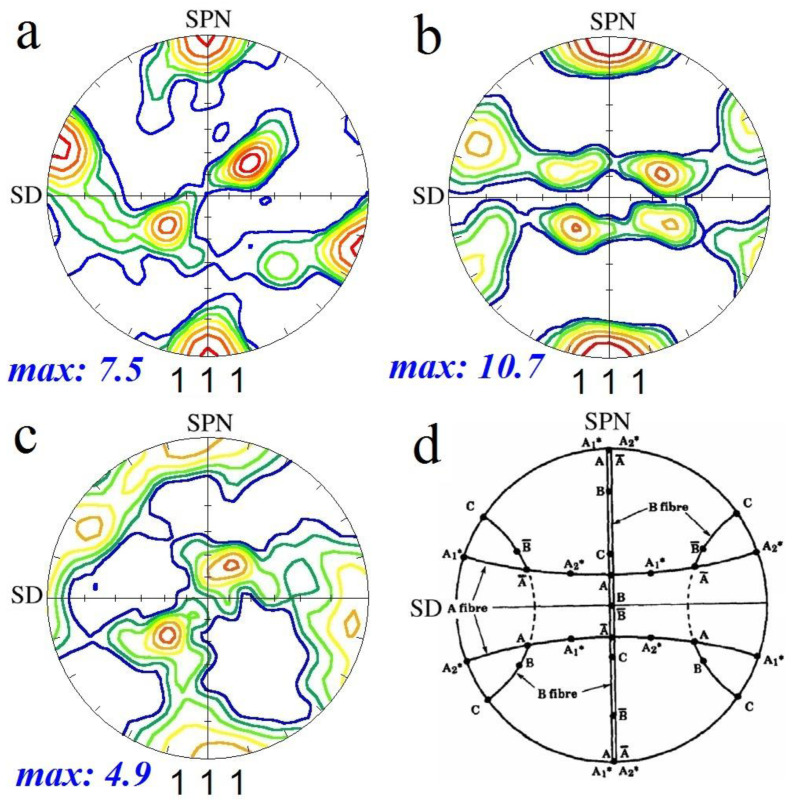
(111) pole figures of different SZs: (**a**) S1, (**b**) S2, and (**c**) S3. SD and SPN denote the shear and shear plane normal directions. (**d**) Ideal simple shear texture components of metals with face-centered crystallographic (FCC) structures [22].

It is known that FSP causes the formation of shear texture components in the SZ [6]. Therefore, for the analysis of texture components in SZs, they are typically compared with simple shear texture components for FCC metals and alloys, as listed in Table 1. However, owing to the complex deformation in the SZ during FSP, the texture components exhibit a mismatch with those in simple shear deformation. To overcome this problem, the as-acquired PFs should be rotated about the X, Y, and Z axes, which aligns them with a standard frame of reference composed of shear and shear plane normal directions (SD and SPN) [6]. In Figure 10, the rotated (111) PFs for S1, S2, and S3 and the ideal simple shear texture components of metals with FCC crystallographic structures are illustrated.

As shown in Figure 10a and Table 1, the first pass of FSP resulted in the formation of the $A_{1}^{*}\left(\left(111\right)\left[\bar{1}\bar{1}2\right]\right)$ shear texture component in the SZ, with an intensity 7.5 times larger than the random texture. The second pass of FSP (Figure 10b) resulted in more intense shear texture components (intensity = 10.7) for both the $A_{1}^{*}\left(111\right)\left[\bar{1}\bar{1}2\right]$ and $A_{2}^{*}\left(111\right)\left[11\bar{2}\right]$. The third pass of FSP (Figure 10c) led to the consumption of $A_{2}^{*}$ and a reduction in the texture intensity to 4.9. This texture analysis reveals that, at the first and second passes of the FSP, the grains inherit the shear texture induced by the rotational tool. By repeating the FSP, the texture intensity increases and other types of shear texture component can be formed. However, the third pass of FSP has a negative effect on texture intensity, which implies that DRX gradually becomes the dominant microstructural mechanism by further increasing the number of FSP passes.

### 3.4. Mechanical Properties

**Figure 11:**
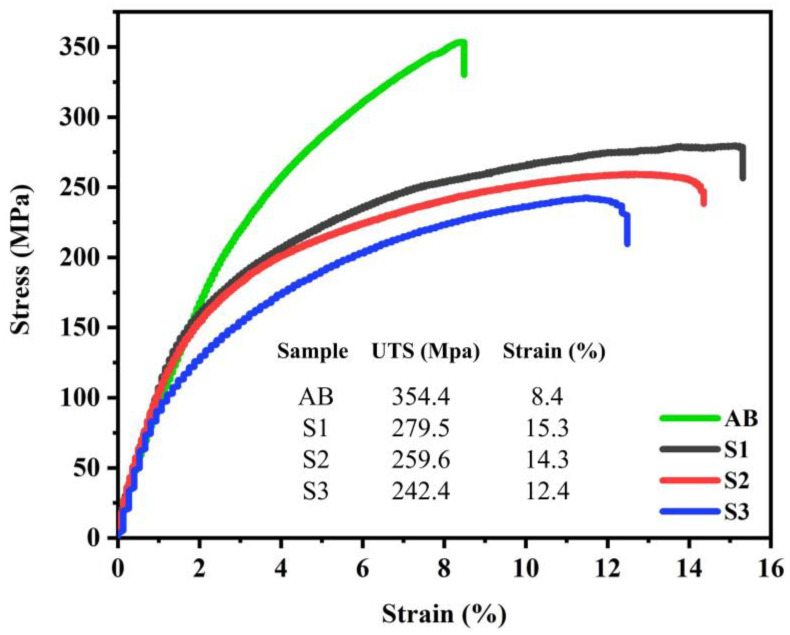
Engineering stress–strain plots of BM and different FSPed samples.

The engineering stress-strain curves of the BM and FSPed samples are illustrated in Figure 11. The results indicated that the as-built BM exhibited high tensile strength (~354 MPa) and low fracture elongation (~8.4%), which is consistent with literature [4,5]. FSP caused a moderate reduction in the ultimate tensile strength (UTS) of the L-PBF AlSi10Mg alloy; however, it resulted in significantly higher fracture elongation in all three passes. Moreover, the first pass of FSP resulted in higher UTS and elongation compared to the second and third passes. It is evident that, by increasing the number of passes, both the UTS and elongation decreased.

**Figure 12:**
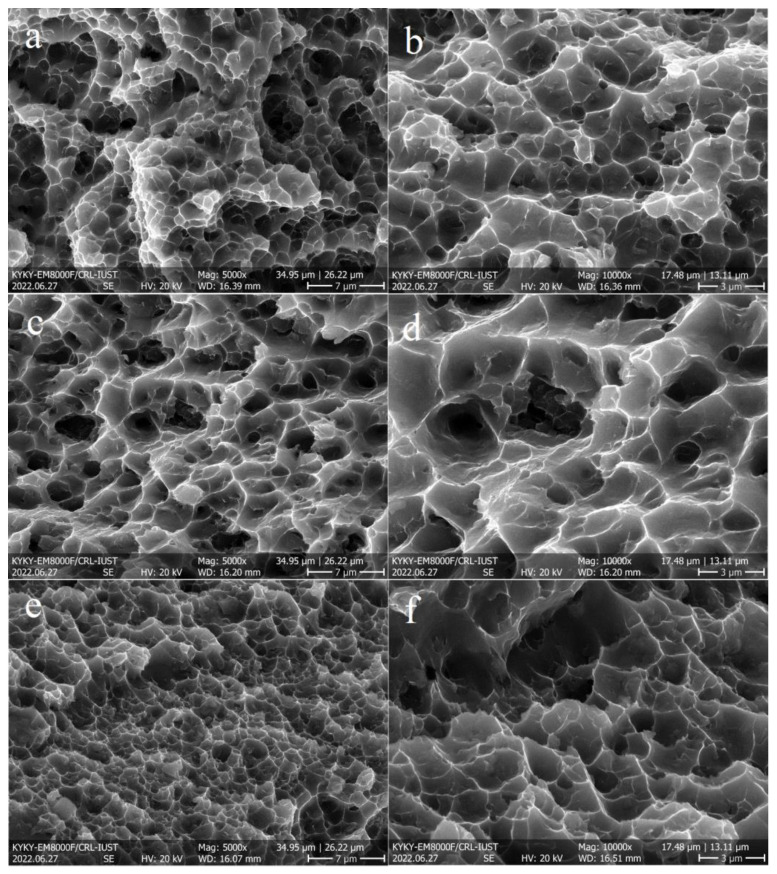
Secondary electron SEM images of fractured surfaces at different magnifications: (**a**,**b**) S1, (**c**,**d**) S2, and (**e**,**f**) S3.

**Figure 13:**
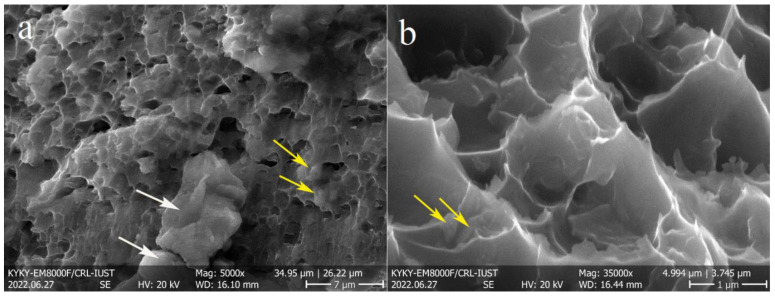
Secondary electron SEM images of fractured surfaces: (**a**) S2, showing the presence of large Si particles, and (**b**) S3, revealing the existence of fine Si particles on the dimple walls.

The SEM images of the FSPed fractured surfaces are illustrated in Figure 12, which confirm the ductile fracture of the samples owing to the presence of dimples. In addition, the fracture surface of S1 (Figure 12a,b) contained finer dimples than those of the other samples (Figure 12c–f), which confirms its higher elongation. Moreover, in all cases, the fractured surface was composed of different-sized dimples, owing to the different grain (Figure 7) and Si particle (Figure 4) sizes in the SZs. Figure 13 revealed the presence of large (white arrows in Figure 13a) and fine (yellow arrows in Figure 13a) Si particles on the fractured surfaces of all samples and interior walls of the dimples (yellow arrows in Figure 13b). Thus, it can be concluded that the damage sites during tensile loading were composed mainly of the AlSi10Mg matrix and, to some extent, Si particles. The correlation between microstructural features and tensile properties is discussed in the next section.

## 4. Discussion

The FSP reduced the strength but enhanced the ductility of L-PBF AlSi10Mg, while one pass of FSP exhibited higher strength and ductility compared to higher numbers (two and three) of FSP passes. The origin of this behavior is discussed in this section. The strength of a polycrystalline metal can be attributed to various mechanisms, such as grain boundary, solid solution, secondary phase or precipitation, dislocation density, and texture-strengthening mechanisms [21]. For brevity, the EBSD data are summarized in Table 2, distinguishing the strengthening mechanisms.

According to Table 2, the as-built BM of the L-PBF AlSi10Mg alloy has a larger grain size (15.8 µm) and lower Taylor factor (2.84) compared to the FSPed samples; however, they exhibit higher strength values (Figure 11). The main reason for the higher strength of the BM is the effect of the eutectic Si networks (Figure 4a), which act as barriers to dislocation movement. The strengthening effect of these Si networks is similar to that of the grain boundaries [23,24]; that is, narrower Si networks are more effective in strengthening the L-BPF AlSi10Mg.

**Figure 14:**
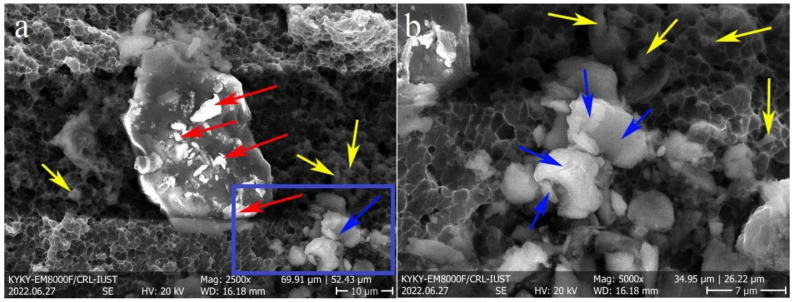
Secondary electron SEM images of the (**a**) fractured surface of FSPed samples, and (**b**) magnified view of the blue rectangle area in (**a**). The red, blue, and yellow arrows refer to the sticking of fine Si particles to the surface of a large one, coalescence of particles, and fine Si particles preset in dimples, respectively.

It is evident from Table 2 that, despite the lower GAM and Taylor factor values of S1, it possesses a higher strength than S2 and S3 (Figure 11). The grain size of S1 is similar to that of S2, and it is larger than that of S3, suggesting that the effect of grain size cannot be the dominant factor on the strength of the FSPed samples. In addition, the same trend was observed in the case of HAGB values. Thus, it can be concluded that the main strengthening mechanism in the FSPed sample is not attributed to those Si particles activated in the Al matrix but is related to their nature. As shown in Figure 4b, the first pass of FSP resulted in the formation of finer Si particles compared to the second and third passes. The finer Si particles are more effective in hindering dislocation movement [25], thus increasing the strength of S1. In the SZ of the S2 and S3 FSPed samples, larger Si particles were formed (Figure 4c,d), indicating Si growth and coarsening. The activation energy for the growth and coarsening of Si particles is the high number of solute Si atoms trapped in the Al matrix because of the high cooling rate during the non-equilibrium solidification of L-PBF AlSi10Mg [15]. During the FSP, the temperature induced by the rotational tool caused the precipitation of supersaturated Si particles. The newly precipitated Si in conjunction with fine Si particles (primarily present in the Al matrix) join together (coalescence mechanism) or join the surface of the larger Si particles, resulting in the continuous growth of Si particles. The fine Si particles (red arrows) on the surface of a large particle and the coalescence mechanism (blue arrows) on the fractured surface of the FSPed samples confirm the suggested growth mechanism (Figure 14). 

**Figure 15:**
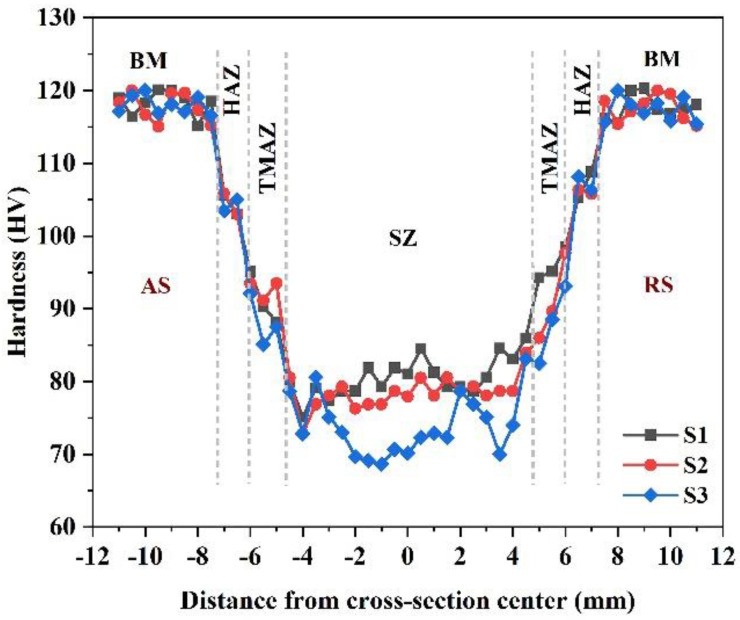
Hardness profiles of BM and different FSPed samples.

This growth mechanism has also been suggested by other studies for the heat treatment of L-PBF AlSi10Mg [2]. More FSP passes provide more time at high temperatures for the diffusion of Si solute atoms, which helps explain the growth mechanism. It is worth noting that the growth of Si particles also has an indirect negative effect on the solid solute strengthening mechanism, as the amount of solute Si atoms is reduced in the Al matrix. For example, energy-dispersive X-ray spectroscopy (EDS) point analysis showed that the amount of solute Si in the matrix was reduced from 2.7 wt.% (for as-built material) to 1.9 wt.% (for the S1 sample). Notably, the lower strength of S3 compared to the other samples is related to the cross-sectional macrostructures (Figure 2), in which the coarse SZ of S3 is larger than that of the other samples. All the tensile samples were broken from the center of the SZs, while for S3 the large coarse SZ adversely affected tensile strength and ductility. This coarse SZ effect was negligible in the cases of S1 and S2 because of the existence of fine SZs in the broken areas of their tensile samples. It is worth noting that the results of hardness measurements confirmed the measured tensile strengths of the samples. The hardness profiles of the samples are illustrated in Figure 15, showing that S1 exhibited the highest SZ hardness values among the samples. In addition, in all cases, FSP induced a sharp reduction in hardness from BM to the SZs. 

**Figure 16:**
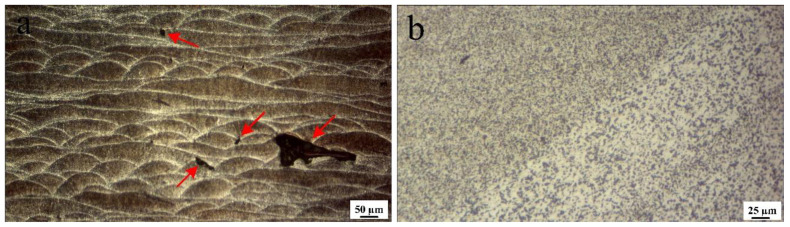
Optical microscope (OM) images of (**a**) large porosities in BM and (**b**) SZ of one-pass FSPed samples showing the absence of large porosities. The red arrows refer to the large porosities in BM (**a**).

The higher ductility of FSPed samples compared to that of the BM (Figure 11) is related to the finer grain sizes of the SZs (Table 2) and elimination of large L-PBF porosities by FSP, as shown in the cross-sectional macrostructures (Figure 2). For more detail, the higher magnified OM images of porosities in BM and their absence after one-pass FSP are shown in Figure 16. 

**Figure 17:**
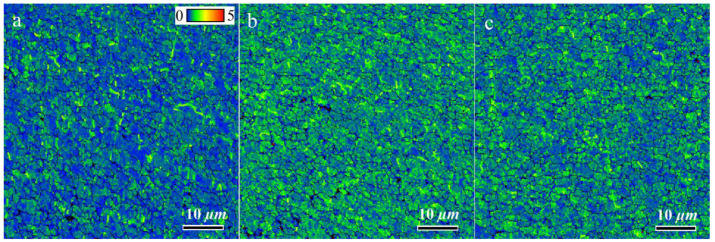
Kernel average misorientation (KAM) maps of SZs in different samples: (**a**) S1, (**b**) S2, and (**c**) S3.

In the case of the FSPed samples, two main reasons can be explored for the higher ductility of S1, as listed in Table 2. Raising the number of FSP passes increased the GAM value from 0.45° (S1) to 0.59° (S2). The higher GAM values indicate a qualitatively higher dislocation density in S2 and S3. Thus, dislocation movement can be hindered owing to their interaction during tensile loading more rapidly than in S1, thus ductility decreases. Notably, the GAM value refers to the effect of the Al matrix on ductility. The kernel average misorientation (KAM) map can be employed to determine the effect of Si particles on ductility. In KAM maps, the misorientation among a grain at the center of the kernel and all points at the border of the kernel is measured. The KAM value of the center point is the average of these misorientations, which indicates the amount of stress concentration [26,27]. The more uniform distribution and lower KAM values indicate less-concentrated stresses in the samples, which causes delayed necking and higher ductility [15]. The KAM maps of the SZs in different samples are illustrated in Figure 17, which shows the existence of the initial concentrated stress in all samples. However, for S1, the KAM values were lower and more uniform than those of the other samples. During tensile loading, the different deformations of the hard Si particles and soft Al matrix cause inhomogeneity in the plastic strain. To accommodate this inhomogeneity, geometrically necessary dislocations (GNDs) are formed, hence a strain gradient or stress concentration will develop in the interfacial zones. The primary high and non-uniform KAM values in S2 and S3 can promote the formation of a strain gradient during tensile loading, hence lower ductility. Finally, from the perspective of the Al matrix, owing to the lower amount of GAM in S1, it has a higher strain-hardening capacity compared to other samples, therefore causing higher ductility.

## 5. Conclusions

Multipass FSP was used to modify the microstructure and mechanical properties of the L-PBF AlSi10Mg plates. The following conclusions were made:

(1) FSP causes the formation of different microstructural zones, such as the BM, HAZ, TMAZ, and SZ, in various metals and alloys, as reported in literature. However, in the case of L-PBF AlSi10Mg, the SZs comprised two distinct fine and coarse regions in accordance with the size of the Si particles. The amount of coarse SZ in the one- and two-pass FSPed samples was negligible. However, in the three-pass FSPed sample, a significant part of the SZ belonged to the coarse SZ.
(2) The elongated grains and BD//[001] texture in the as-built AlSi10Mg disappeared after FSP, and fine equiaxed grains with shear texture components were formed. In addition, the fiber-like Si networks were replaced with Si particles because of their fragmentation during the FSP.
(3) The main reason for the higher strength of the as-built AlSi10Mg compared to those of the FSPed samples is the effect of eutectic Si networks, which act as barriers to dislocation movement, such as the effect of grain boundaries on the strength of metals and alloys based on the Hall–Petch relationship.
(4) The main strengthening mechanisms in the FSPed samples were not related to the Al matrix; however, they were attributed to the Si particles. By repeating the FSP (two and three passes), the Si particles grow by consuming the fine Si particles and Si solute atoms in the Al matrix, resulting in larger and fewer particles. The finer Si particles and higher solute Si atoms in the one-pass FSPed sample led to higher tensile strength compared with the other samples.
(5) Finer grain sizes of SZs and elimination of large L-PBF porosities by FSP are the main reasons for the higher ductility of the FSPed samples compared to that of the as-built AlSi10Mg. The higher ductility of the one-pass FSPed sample can be attributed to the lower and uniform distribution of KAM values and lower GAM values. Notably, in the case of three-pass FSP, the existence of a severely coarse SZ in the macrostructure is the additional origin of lower ductility.
(6) The outcome of this study can be useful for the material science community, particularly for studies investigating the post-treatment of L-PBF AlSi10Mg.

## Figures and Tables

**Table 1:** Ideal texture components with corresponding Euler angles and Miller indices in simple shear deformation of metals with a face-centered cubic (FCC) crystallographic structure [6].

Symbol	Euler Angles (°)			Miller Indices (hkl)<uvw>
	φ_1_	Φ	φ_2_	
$A_{1}^{*}$	35.26/215.26	45	0/90	$\left(111\right)\left[\bar{1}\bar{1}2\right]$
	125.26	90	45	
$A_{2}^{*}$	144.74	45	0/90	$\left(111\right)\left[11\bar{2}\right]$
	54.74/234.74	90	45	
A	0	35.26	45	$\left(1\bar{1}1\right)\left[110\right]$
$\bar{A}$	180	35.26	45	$\left(\bar{1}1\bar{1}\right)\left[\bar{1}\bar{1}0\right]$
B	0/120/240	54.74	45	$\left(1\bar{1}2\right)\left[110\right]$
$\bar{B}$	60/180	54.74	45	$\left(\bar{1}1\bar{2}\right)\left[\bar{1}\bar{1}0\right]$
C	90/270	45	0/90	{001}〈110〉
	0/180	90	45	

**Table 2:** EBSD data of the grain and texture characteristics in the BM and SZs. The data comes to the fine SZs as shown in cross-sectional macrostructures (Figure 2).

Sample	D_av_ (µm)	HAGBs (%)	LAGBs (%)	GAM Value (°)	Taylor Factor	Texture Components	Texture Intensity
BM	15.8	81	19	0.53	2.84	BD//[001]	3.0
S1 (1 pass)	3.0	80	20	0.45	2.98	$A_{1}^{*}$	7.6
S2 (2 pass)	3.1	71	29	0.59	3.12	$A_{1}^{*}$ and $A_{2}^{*}$	10.7
S3 (3 pass)	2.5	83	17	0.54	3.18	$A_{1}^{*}$	4.9

## Data Availability

The raw/processed data required to reproduce these findings cannot be shared at this time as the data also forms part of an ongoing study.
